# Amyloidogenic amyloid-β-peptide variants induce microbial agglutination and exert antimicrobial activity

**DOI:** 10.1038/srep32228

**Published:** 2016-09-14

**Authors:** Philipp Spitzer, Mateja Condic, Martin Herrmann, Timo Jan Oberstein, Marina Scharin-Mehlmann, Daniel F. Gilbert, Oliver Friedrich, Teja Grömer, Johannes Kornhuber, Roland Lang, Juan Manuel Maler

**Affiliations:** 1 Department of Psychiatry and Psychotherapy, Friedrich-Alexander-University Erlangen-Nuremberg, Schwabachanlage 6, D-91054 Erlangen, Germany; 2 Department of Medicine III, Institute for Clinical Immunology, Friedrich-Alexander-University Erlangen-Nuremberg, Gluecksstraße 4a, D-91054 Erlangen, Germany; 3 Electron Devices, Friedrich-Alexander-University Erlangen-Nuremberg, Cauerstraße 6, D-91058 Erlangen, Germany; 4 Institute of Medical Biotechnology, Friedrich-Alexander-University Erlangen-Nuremberg, Paul-Gordan-Str. 3, D-91052 Erlangen, Germany; 5 Institute of Clinical Microbiology, Immunology and Hygiene, Friedrich-Alexander-University Erlangen-Nuremberg, Wasserturmstr. 3/5, D-91054 Erlangen, Germany

## Abstract

Amyloid-β (Aβ) peptides are the main components of the plaques found in the brains of patients with Alzheimer’s disease. However, Aβ peptides are also detectable in secretory compartments and peripheral blood contains a complex mixture of more than 40 different modified and/or N- and C-terminally truncated Aβ peptides. Recently, anti-infective properties of Aβ peptides have been reported. Here, we investigated the interaction of Aβ peptides of different lengths with various bacterial strains and the yeast *Candida albicans*. The amyloidogenic peptides Aβ_1-42_, Aβ_2-42_, and Aβ_3p-42_ but not the non-amyloidogenic peptides Aβ_1-40_ and Aβ_2-40_ bound to microbial surfaces. As observed by immunocytochemistry, scanning electron microscopy and Gram staining, treatment of several bacterial strains and *Candida albicans* with Aβ peptide variants ending at position 42 (Aβ_x-42_) caused the formation of large agglutinates. These aggregates were not detected after incubation with Aβ_x-40_. Furthermore, Aβ_x-42_ exerted an antimicrobial activity on all tested pathogens, killing up to 80% of microorganisms within 6 h. Aβ_1-40_ only had a moderate antimicrobial activity against *C. albicans*. Agglutination of Aβ_1-42_ was accelerated in the presence of microorganisms. These data demonstrate that the amyloidogenic Aβ_x-42_ variants have antimicrobial activity and may therefore act as antimicrobial peptides in the immune system.

Amyloid-β (Aβ) peptides are the main component of the plaques in the brains of patients with Alzheimer’s disease but are also found in healthy individuals^1^,^2^. Amyloid deposits are also observed after intranasal infection of mice with bacteria^3^. In soluble form, Aβ peptides are predominantly located in the cerebrospinal fluid, but these peptides are also generated in most other tissues and are detectable in several bodily fluids, including saliva and urine^4^,^5^. Aβ is a phylogenetically ancient peptide that is highly conserved across species, but its physiological function remains to be elucidated^6^. Aβ peptides are generated via sequential proteolytic cleavage from the membrane-anchored amyloid precursor protein. In addition to the long-known β- and γ-secretases, several other enzymes, such as meprin-β, caspase and aminopeptidase A, are potentially involved in this process^7^,^8^,^9^,^10^. To date, more than 40 different N- and C-terminal truncated Aβ peptide variants consisting of 37 to 43 amino acids have been identified^11^,^12^,^13^.

Due to their structural homology, it has been suggested that Aβ peptides are antimicrobial peptides involved in the innate immune defense system^14^,^15^,^16^. Antimicrobial peptides are peptide antibiotics that act on a variety of Gram-positive and Gram-negative bacteria, fungi and viruses^17^,^18^. Similar to Aβ peptides, antimicrobial peptides are amphiphilic peptides of up to 40 amino acids. They are soluble in aqueous solutions but can also interact with lipid-rich membranes^17^.

In specific conditions, both antimicrobial peptides and Aβ peptides build α-helical structures within pathogen cell membranes, forming ion channels that disturb cell homeostasis and ultimately induce cell death^15^,^19^,^20^,^21^,^22^.

Another antimicrobial function of antimicrobial peptides is pathogen agglutination, which prevents the spread of infection and facilitates phagocytosis^23^. Aggregates of the eosinophil cationic protein—a model for other amyloid-like peptides—induce bacterial agglutination and cell death^24^. Due to their hydrophobic nature, Aβ peptides are prone to aggregation, so their antimicrobial activity may also involve the agglutination of pathogens. Here, we evaluated the antimicrobial and agglutinating activity of several Aβ peptide variants which differ in their C- and N-terminal lengths.

## Materials and Methods

### Cultures of bacteria and fungi

*Enterococcus faecalis* (ATCC 29212), *Listeria monocytogenes* (VA 15110/93), *Escherichia coli* (DH5α) and *Staphylococcus aureus* (ATCC 25923) were grown aerobically on blood agar plates. *Candida albicans* (ATCC 10231) was plated on Sabouraud dextrose agar plates. *E. coli*, *S. aureus* and *C. albicans* were cultured at 37 °C; *E. faecalis* and *L. monocytogenes* were cultured at 37 °C with an additional 5% CO_2_. Before the experiments, the organisms were subcultured to generate mid-logarithmic growth cultures for use as inoculates. Colonies from the agar were transferred using a sterile loop to Mueller-Hinton broth (Roth, Karlsruhe, Germany) and incubated for 100 min at 39 °C to achieve a McFarland density of 0.5. The *C. albicans* and bacterial inocula were normalized in Mueller-Hinton broth to 5 × 10^5^ cells/ml immediately before use. For the experiments, an inoculum of 5 × 10^5^ cells/ml was dispensed into 96-well plates containing Mueller-Hinton broth growth medium with Aβ peptides, as indicated below.

### Peptide pretreatment

Aβ_1-40_, Aβ_1-42_, Aβ_2-40_, Aβ_2-42_ and Aβ_3p-42_ (Anaspec, Fremont, CA, USA) were reconstituted in 1% NH_4_OH (Anaspec, Fremont, CA, USA), diluted with H_2_O_dd_ to reach a final concentration of 1 mg/ml in H_2_O_dd_/0.8% NH_4_OH and stored at −20 °C. Immediately before the experiments, all peptides were diluted in Mueller-Hinton broth to reach a final concentration of 100 μg/ml.

### Flow cytometry

After 6 h of incubation for bacteria and 20 h for *C. albicans*, 50 μl of the cell suspension was diluted in 1% paraformaldehyde (Sigma-Aldrich, Munich, Germany) and stained with DAPI (Sigma-Aldrich, Munich, Germany). Flow cytometry analysis was performed using a Gallios Cytometer (Beckman Coulter, Krefeld, Germany), and the results were evaluated using Kaluza^®^ software (Beckman & Coulter, Krefeld, Germany). Experiments were repeated at least three times for each organism, and duplicates were included for each assay condition. Cultures treated with antibiotics, Mueller-Hinton broth without cells and untreated cells served as controls. Viability was assessed based on the increase in autofluorescence and reduction of forward scatter as signs of microbial damage^25^,^26^.

### Gram staining

The same cultures analysed by flow cytometry were used for the gram staining. After incubating the bacteria and *C. albicans* with the peptides, 10 μl of the cell suspensions were air-dried on microscope slides and flame-fixated. The smears were flooded with crystal violet solution for 2 min. The slides were rinsed and then incubated for 2 min with iodine solution. The slides were then flooded with 96% ethanol for approximately 10 seconds and washed with distilled water. The slides were flooded with safranin for 1 min, washed with distilled water and examined on an Olympus IX 70 microscope (Olympus, Hamburg, Germany) with NIS Elements BR software (Nikon, Duesseldorf, Germany) using a 100× oil immersion objective (1000× magnification).

### Immunocytochemistry

The immunocytochemistry of *E. faecalis* and *S. aureus* was examined using the Gram preparations specified above. To achieve the robust adherence of pathogens on the glass slides during the staining procedure, cells were incubated with 2% paraformaldehyde (Sigma-Aldrich, Munich, Germany) on the slides and air dried for 60 min at room temperature, followed by washing with PBS (Biochrom, Berlin, Germany). Slides were blocked with 1% bovine serum albumin/5% goat serum (Sigma-Aldrich, Munich, Germany) in PBS for 1 h at room temperature before incubation with the Aβ peptide-specific monoclonal antibody 6E10 (Covance, Princeton, New Jersey, USA) at a concentration of 4 μg/ml for 60 min at room temperature. Labeling with the secondary AF488-conjugated goat anti-mouse antibody (Life Technologies, Darmstadt, Germany) at a concentration of 2 μg/ml was performed for 1 h at room temperature. Slides were mounted with Roti^®^-Mount FluorCare DAPI (Roth, Kralsruhe, Germany), and photographs were taken on an automated screening epifluorescence microscope (Eclipse Ti-E, Nikon, Duesseldorf, Germany) equipped with a 100× objective (Plan Apo VC 100× oil, N.A.: 1.4, W.D.: 0.13 mm, Nikon, Duesseldorf, Germany), a motorized stage (TI-S-ER Motorized Stage, Nikon, Duesseldorf, Germany), an sCMOS camera (Neo, Andor, Belfast, UK), a 100-W lamp for illumination (Lambda LS Xenon Arc, Sutter Instrument, Novato, CA, USA) and a filter set (Nikon, Duesseldorf, Germany). Gain and scaling were kept constant throughout all measurements.

### Quantification of Aβ peptides and cell aggregation on the immunocytochemistry slides

**Figure 1:**
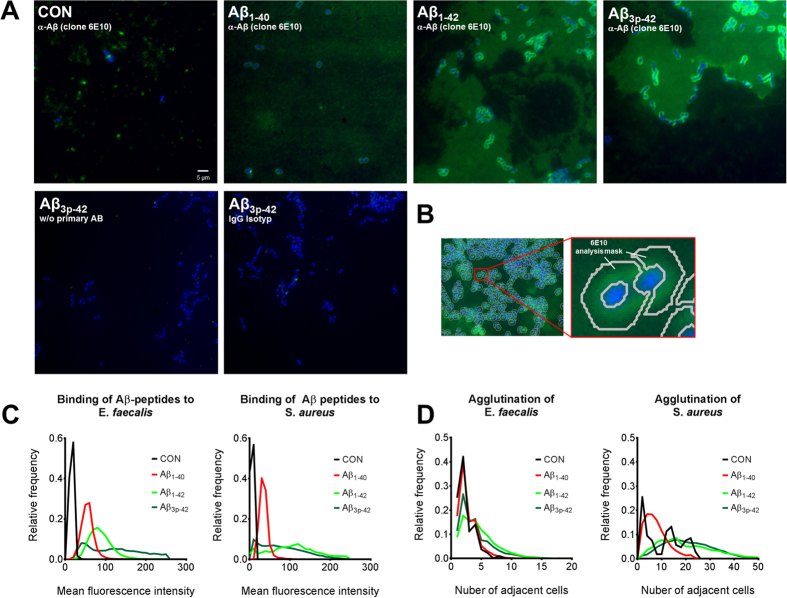
Aβ peptides bind and agglutinate *E. faecalis* and *S. aureus*. Cultures of *E. faecalis* and *S. aureus* were supplemented with 50 μg/ml Aβ_1-40_, Aβ_1-42_, Aβ_1-42_ or Aβ_3p-42_. After 6 h, a sample was transferred to glass slides, flame-fixed and stained with DAPI (blue) and the anti-Aβ monoclonal antibody 6E10 (green) or an IgG-control (**A**). The amount of Aβ peptide bound to *E. faecalis* and *S. aureus* was quantified by calculating donut-shaped masks (**B**) around each bacterium and measuring the mean pixel intensity in this area (**C**). Aggregation was measured by counting the number of cells around each bacterium (**D**). The results are presented as frequency distributions of one representative experiment with *E. faecalis* and one with *S. aureus*; each assay included at least 1,000 bacteria counted in more than 160 images per condition.

All images were visually quality controlled. Images that did not fulfill pre-defined criteria based on image acquisition in the adequate focal plane and adequate cell density were discarded. Of the 2,304 images acquired, 2,072 (~90%) were used for further analysis. An automated quantitative phenotypic image analysis was performed using a custom-adapted version of the image analysis software DetecTiff 27. Pericellular Aβ peptide levels were quantified from the AF488 channel within donut-shaped masks (see Fig. 1B), which were generated from the DAPI channel in a three-step procedure. First, the images of DAPI-stained bacteria were automatically segmented by combined dynamic intensity thresholding and size-dependent particle filtering. In the second step, the binary masks of bacteria were iteratively dilated using a morphological operator to cover the area of the pericellular AF488 signal. Finally, the donut-shaped masks were computed by subtracting the binary masks generated in step one from the masks generated in step two. A total of 57,249 individual donut-shaped analysis masks were constructed from all images. The Aβ peptide level in an individual cell was calculated as the arithmetic mean of all pixel values within the area of a donut-shaped mask and reported as the mean pixel intensity. Cell aggregation was quantified in an automated manner from segmented images of DAPI-stained nuclei by counting the number of bacteria adjacent to a selected individual bacterium within a rectangular 50-pixel^2^ region.

### Scanning electron microscopy of *Enterococcus faecalis*

The specimens were attached to aluminum holders and sputtered with platinum in 15-nm layers using a LEICA EM SCD-500 (Leica, Wetzlar, Germany). The surface morphology of the platinum sputter*-*coated samples was investigated using a JSM 6 610 scanning electron microscope (JEOL, Peabody, MA, USA) at a working distance of 10 mm and an acceleration voltage of 10 kV at room temperature.

### Assessment of proliferation and viability

An inoculum of 5 × 10^5^ cells/ml was dispensed into 96-well plates containing Mueller-Hinton broth growth medium and 3.13 μg/ml or 6.25 μg/ml Aβ-peptides or LL-37 (Anaspec, Fremont, CA, USA). Bacterial plates were incubated aerobically at 37 °C for 6 h, and *C. albicans* was cultured for 20 h at 37 °C. To assess proliferation, 10 μl Alamar Blue^®^ reagent (Life Technologies, Darmstadt, Germany) was added to each well containing a 100-μl sample, followed by incubation for 60 min at 37 °C in the dark. The resulting absorbance was measured with a SpectraMax 340 PC 384 microplate reader (Molecular Devices, Biberach, Germany). The proliferation of *C. albicans* was assessed by measuring turbidity at 570 nm.

The viability of *E. faecalis*, *E. coli* and *C. albicans* was further examined by plating Aβ-treated cultures on agar plates. Cultures of *E. coli*, *E. faecalis* and *C. albicans* were prepared as described above. After incubation with 50 μg/ml Aβ_1-40_, Aβ_1-42_ or Aβ_3p-42_ for 6 h for bacteria and 20 h for *C. albicans*, the cell suspensions were diluted in Mueller-Hinton broth, and the microbial load was determined using an Eddy Jet Spiral Plater (IUL Instruments, Germany) by depositing 50 μl of sample on a rotating agar plate. Bacteria were grown on Mueller-Hinton agar plates, and *C. albicans* was grown on Sabouraud dextrose agar plates. After 24 h of incubation, colonies were counted and translated to colony-forming units (CFU)/ml.

### Assessment of Aβ peptide aggregation

Thioflavin T was used to monitor the aggregation of Aβ_1-42_ in the presence of microorganisms. In a black 96-well plate, 160 μg/ml of Aβ_1-42_ in PBS/0.4% NH_4_OH were mixed with heat inactivated *E. coli*, *S. aureus* or *C. albicans* at a final density of McFarland 0.5. Leaving away the microorganisms or adding 10 μg/ml carboxylated microbeads (Micromod, Rostock, Germany) with a diameter of 0.5 μm served as control. After adding Thioflavin T (Sigma-Aldrich, Munich, Germany) in final concentration of 0.2 mM, kinetic measurement was performed at 37 °C with excitation at 450 nm and emission at 484 nm in a CLARIOstar^®^ microplate reader (BMG labtech, Ortenberg, Germany). Measurements containing the respective microorganisms but no Aβ_1-42_ served as blank.

### Statistical analysis

The statistical analysis was performed using GraphPad Prism^®^ 6.0 software (GraphPad, La Jolla, CA, USA). Although normality tests could not be calculated, due to the small sample sizes, a Gaussian distribution of the data can be assumed. No pairing of the experiments was assumed. Therefore, one-way ANOVA, followed by the Dunnett’s post-test for multiple comparisons against the control were calculated.

## Results

### Aβ peptides bind to bacterial surfaces

*E. faecalis* and *S. aureus* were cultivated in the presence of the Aβ peptide variants Aβ_1-40_, Aβ_2-40_, Aβ_1-42_ and Aβ_3p-42_ at a concentration of 50 μg/ml. After 6 h, the bacteria were smeared on glass slides, labeled with the Aβ peptide-specific mouse monoclonal antibody 6E10 and counterstained with DAPI. Immunofluorescence microscopy revealed Aβ peptide-specific staining on the surfaces of both, *E. faecalis* and *S. aureus* (Fig. 1A). Automated quantification of experiments with *E. faecalis* and with *S. aureus* including more than 1,000 bacteria per condition revealed that Aβ_x-42_ bound more strongly to the surface of the microorganisms than did Aβ_1-40_ (Fig. 1A,C). Aβ-reactive material was also observed in between the bacteria after incubation with Aβ_1-42_ and Aβ_3p-42_, but the intensity was strongest directly around the bacteria.

### Aβ peptides agglutinate microorganisms

Automated analysis of the immunocytochemistry slides further revealed that treatment with Aβ_1-42_ and Aβ_3p-42_ led to agglutination of *E. faecalis* and *S. aureus*. By contrast, incubation with Aβ_1-40_ resulted in almost no agglutination (Fig. 1A,D).

**Figure 2:**
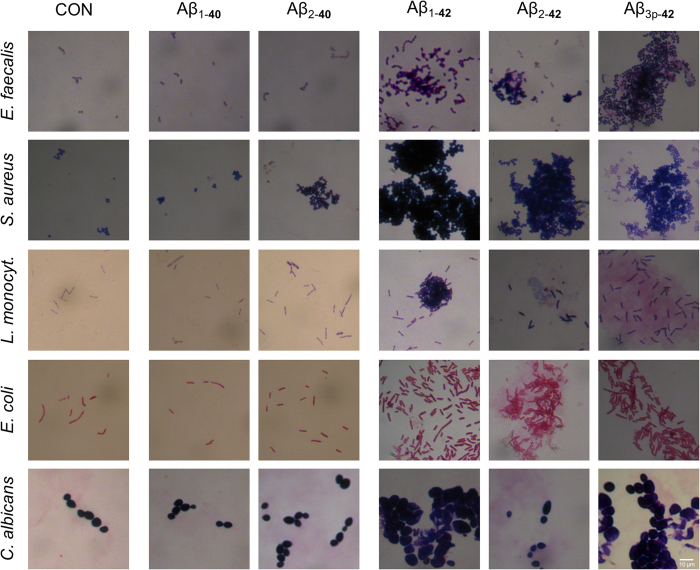
Aβ_x-42_ but not Aβ_x-40_ agglutinates several microorganisms. Gram stains of *E. faecalis, S. aureus, L. monocytogenes, E. coli* and *C. albicans* were prepared after 6 h of incubation (20 h for *C. albicans*) with the indicated Aβ peptide variants at a concentration of 50 μg/ml. Large clusters of agglutinated microorganisms were observed only in cultures treated with Aβ_x-42_. Within the clusters of agglutinated microorganisms, large amounts of amorphic material are evident.

To extend these findings to other pathogens and Aβ peptide fragments, four different bacteria (*E. faecalis*, *E. coli*, *S. aureus* and *L. monocytogenes*) and one fungus (*C. albicans*) were cultured in the presence of the Aβ peptide variants Aβ_1-40_, Aβ_1-42_, Aβ_2-40_, Aβ_2-42_ and Aβ_3p-42_. Gram staining revealed Aβ peptide variant-dependent agglutination of all microorganisms. Neither Aβ_1-40_ nor Aβ_2-40_ induced microbial agglutination, whereas Aβ_x-42_ agglutinated the microorganisms into large clusters (Figs 1 and 2).

**Figure 3:**
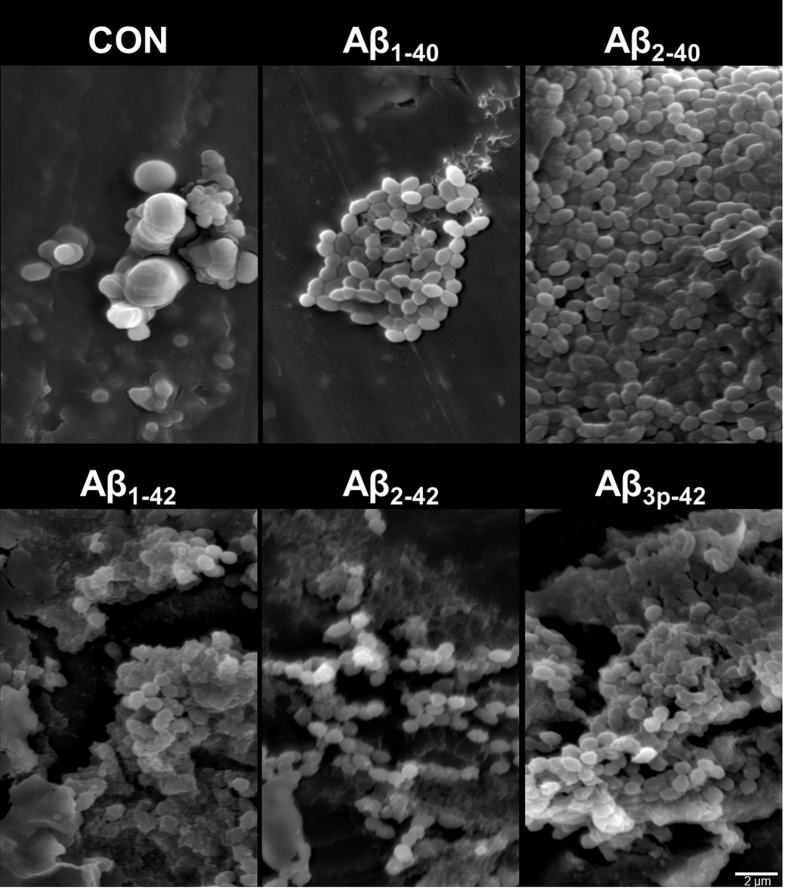
*E. faecalis* agglutinated by Aβ_x-42_ exhibits an irregular, dysmorphic shape and the accumulation of large amounts of amorphic material between cells. Scanning electron microscopy of *E. faecalis* after incubation for 6 h with the indicated Aβ peptide variants at a concentration of 50 μg/ml. The scale bar represents 2 μm.

Scanning electron microscopy of *E. faecalis* incubated with the different Aβ peptide variants confirmed the agglutinating activity of Aβ_x-42_. Additionally, bacteria treated with Aβ_x-42_ were dysmorphic, and large amounts of amorphous material were present within the aggregates. In cultures of *E. faecalis* incubated with Aβ_2-40_, there were larger aggregates of bacteria that retained their vital morphology. Only minor aggregates were observed in the absence of Aβ peptides and in cultures incubated with Aβ_1-40_. Taken together, these results suggest that Aβ_x-42_ peptides induce agglutination of microorganisms (Fig. 3).

### Aβ peptides exert antimicrobial activity

Flow cytometry analysis of the microorganisms treated with Aβ_x-42_ peptides revealed a population characterized by increased autofluorescence at 525 nm and/or reduced forward scatter (AF+/FSC−). Increased autofluorescence and reduced forward scatter are common features of damaged cells^25^,^26^. This population was therefore categorized as damaged microorganisms. DAPI staining was performed to differentiate the microorganisms from the background, and only DAPI-positive events were gated for further analysis. The microbial aggregates observed via immunocytochemistry, Gram staining and scanning electron microscopy were only partially detected by flow cytometry; Compared with the size of untreated and consequently unaggregated microorganisms Aβ peptide treated microorganisms did not show increased forward or side-scatter characteristics.

**Figure 4:**
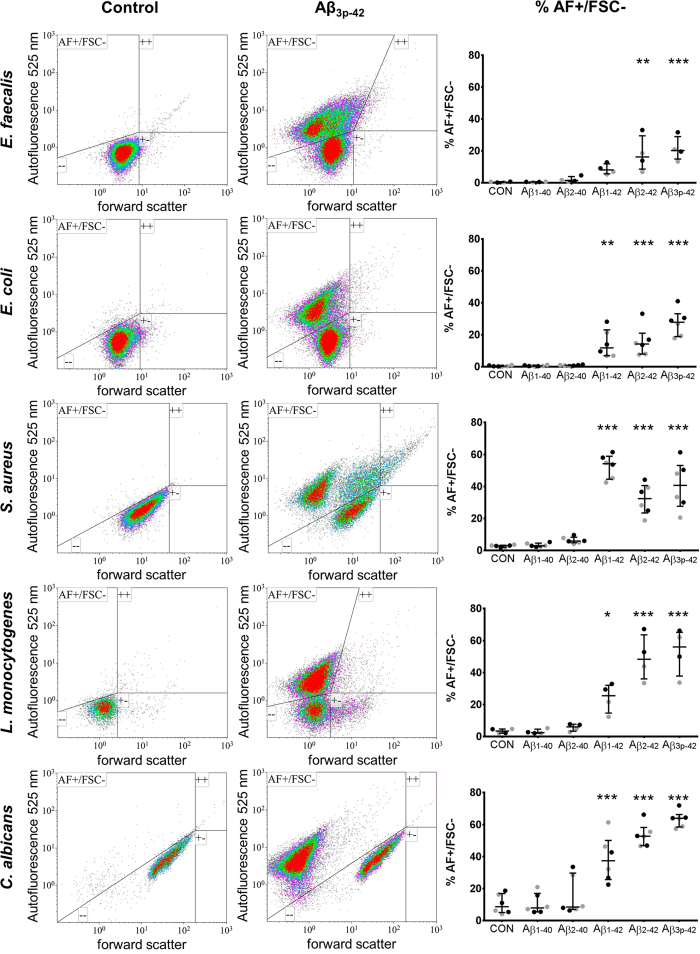
Antimicrobial activity of Aβ_x-42._ Flow cytometry analysis of *E. faecalis, E. coli*, *S. aureus*, *L. monocytogenes* and *C. albicans* after 6 h of incubation with the indicated Aβ peptide variants at a concentration of 25 μg/ml (gray) or 50 μg/ml (black). Column one (control) depicts representative density plots of forward scatter vs. autofluorescence at 525 nm in untreated microorganisms gated for DAPI positivity. Column two (Aβ_3p-42_) shows the same cultures after incubation with Aβ_3p-42_. Column three (% AF+/FSC−) shows the percentage of microorganisms with increased autofluorescence at 525 nm or reduced forward scatter (upper left quadrant) which are supposed to be damaged. The results are depicted as scatter plots with the median ± interquartile range. Cultures treated with 25 μg/ml and 50 μg/ml Aβ-peptides were grouped for the statistical analysis. Asterisks indicate significant differences calculated by the Friedman test followed by Dunn’s post-test. *p < 0.05, **p < 0.01, ***p < 0.001.

Aβ_1-42_ exerted antimicrobial activity on all tested microorganisms. As characterized by reduced forward scatter and increased autofluorescence, damaged microorganisms represented up to 70% of cells in the analyzed samples. N-terminal truncation and pyroglutaminylation of Aβ_1-42_ further increased microbicidal activity against all microorganisms except *S. aureus* (Fig. 4). No microbicidal activity was observed for Aβ_1-40_ or Aβ_2-40_. Identical concentrations of Aβ peptides did not agglutinate human THP-1 cells and were not toxic to those cells within 24 h.

**Figure 5:**
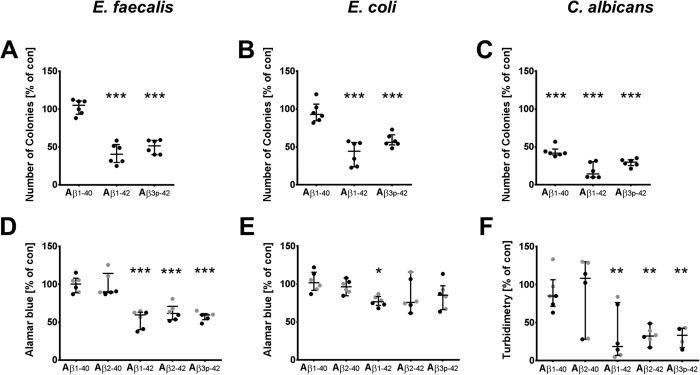
Aβ_x-42_ reduces the number of colony-forming units and proliferation of microorganisms. Colony forming units (CFU) of *E. faecalis*, *E. coli* and *C. albicans* were determined after treatment with 50 μg/ml Aβ_1-40_, Aβ_1-42_ and Aβ_3p-42_ for 6 h (24 h for *Candida*) by plating the cultures on agar plates and incubating for 24 h (**A–C**). The proliferation of the bacteria incubated with the indicated Aβ peptides at a concentration of 6.25 μg/ml (black) or 3.13 μg/ml (grey) was assessed by the Alamar Blue assay after 6 h (**D,E**). The proliferation of *C. albicans* was assessed by turbidimetry after incubation for 24 h (**E**). The results are expressed relative to untreated cultures and are depicted in a scatter plot together with the median and interquartile range. Asterisks indicate significant differences calculated by the Friedman test followed by Dunn’s post-hoc test. *p < 0.05, **p < 0.01, ***p < 0.001.

The antimicrobial activities of Aβ_1-42_ and Aβ_3p-42_ were further confirmed by the Alamar blue test for bacteria, by turbidimetry for *Candida* and by seeding the bacterial and fungal cultures on agar plates (Fig. 5). Incubating *E. coli* and *E. faecalis* with 50 μg/ml Aβ_1-42_ or Aβ_3p-42_ reduced the number of colony forming units by 50% compared to untreated cultures (Fig. 5A,B). The number of colony forming units was not reduced by Aβ_1-40_. The toxic effects of the Aβ peptides were even stronger against *C. albicans*. Incubation with Aβ_1-42_ or Aβ_3p-42_ reduced the number of colony forming units by 85% (Fig. 5C). Aβ_1-40_ also had a moderate antimicrobial effect against *C. albicans* in culture.

### Agglutination of Aβ_1-42_ is accelerated by microorgansims

**Figure 6:**
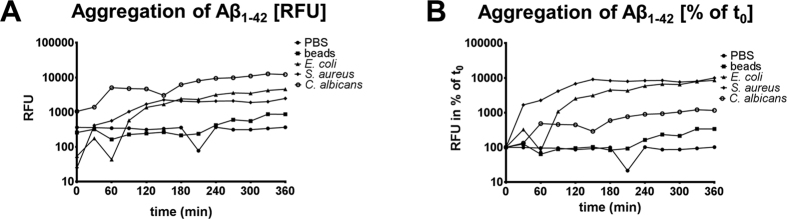
Microorganisms accelerate the agglutination of Aβ_1-42_. Thioflavin fluorescence (Ex 450 nm, Em 484 nm) was monitored for 6 h at 37 °C in solutions of 160 μg/ml Aβ_1-42_ in PBS. Each measurement was corrected for the fluorescence in absence of Aβ_1-42_. Blank corrected Relative fluorescence units (RFU) are depicted in (**A**) whereas the change of RFU in relation to the first measurement at t_0_ is graphed in (**B**).

The agglutination of Aβ_1-42_ in PBS was monitored by the Thioflavin assay over 6 h at 37 °C. Adding heat inactivated *E. coli*, *S. aureus* or *C. albicans* resulted in a more rapid increase in Thioflavin fluorescence than agglutination of Aβ_1-42_ in PBS alone or in the presence of carboxylated polystyrene microbeads with a diameter of 0.5 μm. This difference was obvious in terms of relative fluorescence units (RFU, Fig. 6A) or when expressed in relation to fluorescence at the beginning of the measurement (% of t_0_, Fig. 6B).

## Discussion

We observed that the more amyloidogenic Aβ_x-42_ peptides led to the agglutination and death of microorganisms. For Aβ_1-40_ only an antifungal activity was seen.

Microbial agglutination induced by Aβ_x-42_ was observed by Gram staining, immunocytochemistry and scanning electron microscopy. Microbial agglutination was accompanied by binding of the Aβ peptide to the microbial surface. Only in cultures of *L. monocytogenes* and *C. albicans* no agglutinated bacteria were observed after the treatment with Aβ_2-42._ As there can still be seen this red amorphic material, it is well possible that the bacteria already disintegrated and left only debris. This is supported by the fact that a very strong microbicidal effect for Aβ_2-42_ is also observed by flow cytometry. Also, when looking at the gram stains of Aβ_3p-42_ treated *L. monocytogenes* it seems that there are already several bacteria “missing” within the agglutinate. The antimicrobial peptides eosinophil cationic protein and salivary agglutinin^23^,^24^ also induce agglutination. The agglutination of pathogens by Aβ-peptides may contribute to antimicrobial control via several mechanisms. First, agglutination may prevent the distribution of microorganisms by causing physical immobilization. Second, agglutination facilitates phagocytosis^23^,^24^. Third, Aβ peptides act as opsonins for phagocytosis^28^.Thereby, the Aβ peptide variants with the highest microbicidal activity are also the most effective in inducing phagocytosis^28^. Finally, in addition to bacterial agglutination, Aβ_x-42_-peptides have direct antimicrobial activity. Microorganisms exposed to Aβ_x-42_ exhibited reduced forward scatter and increased autofluorescence in flow cytometry analyses. A loss of forward scatter is a common feature of damaged cells and bacteria, but increased autofluorescence of bacteria exposed to bactericidal agents has only recently been observed^25^. Renggli *et al.* suggested that this autofluorescence is caused by a change of cell morphology^25^.

Autofluorescence after excitation with ultraviolet and blue light was also observed in amyloid plaques containing full length Aβ peptides and after aggregation of synthetic Aβ peptides^29^,^30^,^31^. As can be seen from the IgG staining control in Fig. 1, the detected signals do not result from autofluorescence. In the flow cytometry experiments, the reported autofluorescence after treatment with Aβ peptides also occurred after treatment with antibiotics (data not shown). Therefore we suppose, that autofluorescence in our experiments is due to cell death. However, we cannot fully exclude, that part of the observed fluorescence is due to autofluorescence of Aβ peptides.

The antimicrobial activity of amyloidogenic Aβ peptides was confirmed by reduced Alamar blue turnover and a reduction in colony forming units in our study. In case of *E. faecalis*, both assays showed a consistent reduction of bacterial growth by the Aβ_x-42_ peptides. In contrast, the decrease in *E. coli* CFU by these peptides was paralleled by a weaker effect on Alamar blue turnover (Fig. 5B,E), indicating that agglutination of *E. coli* may lead to overestimation of the killing activity in the CFU assay. On the other hand, the Alamar blue test and turbidimetry might have underestimated the antimicrobial effect, as the microbial aggregates could disturb the optical path, leading to heterogeneous and false high readings. This could explain, why we miss the cytotoxic effect of Aβ_1-40_ and Aβ_2-40_ on *C. albicans* with the Alamar blue assay.

C- and N-terminal modifications of the Aβ peptides greatly affected their aggregation and antimicrobial activity, such that Aβ_x-42_ was much more effective than Aβ_x-40_. N-terminal truncation and pyroglutaminylation further enhanced the antimicrobial activity. The differences in the effects of the Aβ peptide variants are most likely due to their physicochemical characteristics. Aβ_x-42_ is much more hydrophobic than Aβ_x-40_. The truncated and pyroglutaminylated N-terminus further enhances this hydrophobicity^32^,^33^,^34^,^35^. The increased hydrophobicity of Aβ_x-42_ peptides is also reflected by their increased binding to the surface of the microorganisms. Therefore, there is a good correlation between Aβ-peptide hydrophobicity and the binding/agglutination of microorganisms. While the exact mechanism by which Aβ-peptides kill microorganisms remains to be elucidated, it seems as if the heparin binding domain of the Aβ peptide sequence is involved. Mannan and glucan are suggested to bind to Aβ peptides via its heparin-binding site^36^,^37^,^38^. By competitive binding of the heparin binding site by mannan and glucan, the agglutination and binding of Aβ peptides to microbes was effectively inhibited^36^. Furthermore, binding of the heparin binding site of Aβ peptides promotes their aggregation and the formation of β-sheet structures^39^,^40^. This explains, why we found an accelerated aggregation of Aβ_1-42_ when incubated together with *E. coli*, *S. aureus* and *C. albicans*. Binding of proteoglycans to the heparin-binding site of Aβ peptides has been suggested as an early step in plaque formation^39^. Eosinophil cationic protein is also an amyloid-like protein. Its antimicrobial activity depends on its ability to agglutinate bacteria and form amyloid fibrils on the bacterial surface^24^. The eosinophil cationic protein mediated agglutination of bacteria is followed by membrane leakage and cell death^24^.

The antimicrobial activity of Aβ_x-42_-peptides demonstrated in this study is consistent with previous reports^16^,^36^. However, Soscia *et al.* found, that Aβ_1-40_ is not as effective as Aβ_1-42_ but still possesses antimicrobial activity against bacteria and fungi. This effect strongly differed between the investigated microorganisms and was in several bacteria near the limit of detection. In respect to the colony forming units, we only observed an antimicrobial activity of Aβ_1-40_ against *C. albicans.* In accordance with the report of Kumar *et al.*, this activity was weaker as for the Aβ_x-42_ peptides. The discrepancy concerning the effect against bacteria might be due to the selection of different bacterial strains or differences in Aβ peptide pretreatment. Aβ peptides are prone to self-aggregation, and the method of synthesis, the solvents used and the time until application strongly affect their conformation and, consequently, their biological activity. Thus, while an antifungal effect of Aβ_1-40_ was consistently shown, its impact against bacteria needs to be further investigated. Having observed increased phagocytotic activity of monocytes after stimulation with Aβ_2-40_, we previously suggested, that the more soluble Aβ_x-40_ peptides, may also act as auto- or paracrine factors regulating immune activity^28^.

The physiological relevance of Aβ peptide antimicrobial activity is supported by several lines of evidence.

- In an experimental model of meningitis, survival was reduced in mice that were not able to produce Aβ peptides due to a knockout of its precursor. Reciprocally, mice overexpressing Aβ peptides showed reduced mortality in this model^36^.
- Acute infections of the brain result in reduced Aβ_42_ levels (but not Aβ_40_) in cerebrospinal fluid but increased deposition in brain tissue^3^,^41^,^42^,^43^,^44^.
- Aβ peptide deposition has also been reported in chronic infections of the central nervous system, such as neuroborreliosis, neurosyphilis, HIV or Herpes simplex encephalitis^22^,^45^,^46^,^47^. Recently, Diana Pisa and her colleagues observed *Candida* species in brain regions affected by Alzheimer’s disease pathology but not in normal controls^48^. In our study, Candida was particularly sensitive to Aβ-peptide-induced aggregation and cell death.
- The γ-secretase blocker DAPT impairs recovery from lipopolysaccharide-induced inflammation in the rat brain^49^.
- Monocytes, microglia and astrocytes increase the expression of amyloid precursor protein and release Aβ peptides upon activation by lipopolysaccharide^50^,^51^,^52^. In several mouse models, lipopolysaccharide induces amyloid precursor protein expression and subsequent Aβ plaque deposition^22^.

These findings suggest that the secretion of Aβ-peptides is part of the innate immune defense in the CNS. Aβ-lowering therapies might therefore hamper the resistance of the brain to infections and malignant disorders. The increased neonatal mortality of BACE1/BACE2 knock-out mice only in animals housed under non-sterile conditions supports this assumption^53^. Special attention should therefore be given to these events during clinical tests of Aβ-lowering therapies. Others have even suggested that an infectious agent may be involved in Alzheimer’s disease pathogenesis^22^,^46^,^54^. Our finding of accelerated Aβ_1-42_ agglutination in the presence of microorganisms may support this hypothesis. Further research is needed to determine whether Aβ plaque deposition is the consequence of a microbial infection or a mechanism of CNS immune defense that is misled in Alzheimer’s disease.

## Additional Information

**How to cite this article**: Spitzer, P. *et al.* Amyloidogenic amyloid-β-peptide variants induce microbial agglutination and exert antimicrobial activity. *Sci. Rep.* **6**, 32228; doi: 10.1038/srep32228 (2016).
